# Genome-Wide Knockout Screen Identifies EGLN3 Involving in Ammonia Neurotoxicity

**DOI:** 10.3389/fcell.2022.820692

**Published:** 2022-03-29

**Authors:** Jiequn Li, Chunli Chen, Chenchen Li, Zhiping Hu, Jieqiong Tan, Liuwang Zeng

**Affiliations:** ^1^ Department of Liver Transplant, Second Xiangya Hospital, Central South University, Changsha, China; ^2^ Department of Neurology, Second Xiangya Hospital, Central South University, Changsha, China; ^3^ Center for Medical Genetics, School of Life Sciences, Central South University, Changsha, China; ^4^ Hunan Key Laboratory of Medical Genetics, Central South University, Changsha, China; ^5^ Hunan Key Laboratory of Animal Model for Human Diseases, Central South University, Changsha, China

**Keywords:** hepatic encephalopathy, ammonia, clustered regularly interspaced short palindromic repeats/CRISPR-associated protein 9, egl-9 family hypoxia-inducible factor 3, mitochondrial apoptosis

## Abstract

Hepatic encephalopathy (HE) is a brain dysfunction associated with poor quality of life, increased morbidity and mortality. The pathogenesis of HE is still not fully clarified and effective therapeutic strategies are imperative. Among multiple factors that contribute to the pathophysiological process of HE, ammonia neurotoxicity is thought to be central in the pathogenesis of HE. Therefore, in this study, we subjected SH-SY5Y cells to ammonia insult and performed a pooled genome-wide CRISPR (clustered regularly interspaced short palindromic repeats)/Cas9 (CRISPR-associated protein 9) knockout screen to unveil the underlying molecular mechanisms of ammonia neurotoxicity and discover new potential therapeutic targets for HE. We found that EGLN3 (egl-9 family hypoxia-inducible factor 3) UCP3,GTPBP5, OR4D11 and SDR9C7 with 6 unique sgRNAs may contribute to protection against ammonia injury, while EGLN3 may be most related to ammonia resistance. We knocked down EGLN3 by transfecting neurons with specific shRNA lentivirus and confirmed that EGLN3 knockdown decreased ammonia-induced caspase-3 activation and apoptosis. We also demonstrated that EGLN3 knockdown ameliorated ammonia induced decreased expression of Bcl-2, increased expression of Bax and inhibited release of cytochrome c into the cytosol in neurons, suggesting that EGLN3 inhibition protected against ammonia induced apoptosis through mitochondrial dependent apoptosis pathway. Future therapeutic strategies regulating EGLN3 may be applied to the management of HE.

## Introduction

Hepatic encephalopathy (HE) is a brain dysfunction associated with both acute or chronic severe liver diseases. It characterized by a wide range of neuropsychiatric abnormalities (Wijdicks, 2016). The pathogenesis of HE is still not fully clarified. Multiple factors have been proposed to contributed to the pathophysiological process of HE, including excessive formation of ammonia, impaired brain energy metabolism (Butterworth, 2014; Haussinger et al., 2021), inflammation (Coltart et al., 2013), neuronal autoantibody elevation (Manzhalii et al., 2021), autophagic flux in astrocytes (Lu et al., 2019) and specific gut microbial taxa (Ahluwalia et al., 2016). HE is associated with poor quality of life, increased morbidity and mortality. It has brought a heavy clinical and economic burden to the society. Therefore, effective therapeutic strategies for HE are imperative.

Among multiple pathogenic factors, ammonia is thought to be central in the pathophysiology of HE. Ammonia impairs hemichannel-mediated lactate transport between neurons and astrocytes and leads to neuronal energy deficit in HE (Hadjihambi et al., 2017). Hyperammonemia impairs mitochondrial energy metabolism in astrocytes rapidly in a GDH2- dependent manner (Drews et al., 2020). Ammonia also impairs astroglial Slc38 glutamine transporters and contributes to neuropsychiatric abnormalities in acute liver failure (Hamdani et al., 2021). Ammonia-lowering approaches are the main strategies for the prevention and treatment of HE. Phosphatidylserine administration can reduce blood ammonia and neuropsychiatric abnormalities through encountering endotoxin (Zamanian et al., 2020). However, the exact molecular mechanisms of ammonia in HE remain poorly understood. A better understanding of the underlying mechanisms will pave way to developing novel therapeutic strategies for HE.

The clustered regularly interspaced short palindromic repeats/CRISPR-associated protein 9 (CRISPR/Cas9) is an efficient and simple tool for gene editing and has been extensively used in biomedicine, basic research and biotechnology in various species and cell types (Hryhorowicz et al., 2017). Genome-scale CRISPR-Cas9 knockout (GeCKO) screening is a promising way to interrogate gene function on a genome-wide scale (Shalem et al., 2014), which would help us to explore the molecular mechanisms of disease processes and discover new therapeutic targets (Gupta et al., 2019). To explore the molecular mechanisms underlying ammonia induced neurotoxicity, we employed GeCKO screening technology to identify new regulators involved in ammonia induced neurotoxicity in SH-SY5Y cells. EGLN3 (egl-9 family hypoxia-inducible factor 3) was validated as a novel ammonia resistance gene in this study and it is a potential therapeutic target for ameliorating ammonia induced neurotoxicity in HE.

## Materials and Methods

### Lentiviral Production of the sgRNA Library

Lentivirus of sgRNA library was prepared following the protocol previously described (Cai et al., 2019). Briefly, HEK293T cells were divided into a 10 cm dish and cultured in DMEM supplemented with 10% fetal bovine serum (FBS). GeCKO library (#1000000048, Addgene, Watertown, MA, United States), pV-SVg (#8454, Addgene) and psPAX2 (#12260, Addgene) packing plasmids were co-transfected at a mass ratio of 1:0.5:1.5 using Lipofectamine 3000 reagent (Invitrogen, San Diego, CA, United States) following the manufacturer’s protocol. 6 h after transfection, the cell culture media was replaced with fresh medium. The cell supernatant was collected and cell debris was removed by centrifugation at 3,000 rpm at 4°C for 30 min. After centrifugation, the supernatant was filtered with a 0.45-µm pore size filter and then ultracentrifuged at 24,000 rpm for 2 h at 4°C for concentration. Concentrated virus was resuspended with PBS overnight at 4°C, and stored at −80°C refrigerator.

### Infection of the sgRNA Lentiviral Library

SH-SY5Y cells were cultured in the DMEM containing 10% FBS (Invitrogen, San Diego, CA, United States) with 4 mM L-glutamine at 37°C, 5% CO_2_. 3 × 10^8^ SH-SY5Y cells were infected with the GeCKO library viruses at a multiplicity of infection (MOI) of 0.1–0.3 to ensure that most cells receive only one viral construct to knockout a single gene. 48 h after infection, SH-SY5Y cells were selected by 1 μg/ml puromycin for 7 days.

### Ammonia Resistance Gene Screening and DNA Sequencing

SH-SY5Y cells were treated with 5 mM ammonium chloride for 24 h in 95% air, 5% CO_2_. The surviving cells were collected. Genomic DNA was extracted for subsequent PCR reaction to amplify sgRNA sequence using a Blood & Cell Culture DNA Midi Kit (Qiagen, Hilden, Germany). PCR was performed following the protocol as described by Dr. Feng Zhang. The amplicons were added by the second PCR and sequenced using a HiSeq 2500 (Illumina, San Diego, CA, United States). The forward primer was 5′-CTT​GTG​GAA​AGG​ACG​AAA​CA-3′. The reverse primer was 5′-GCC​AAT​TCC​CAC​TCC​TTT​CA-3′. The processed data were removed the sequences from beginning to sgRNA priming site primers. Trimmed reads were mapped to the indexed GeCKO v2 libraries A and B. Read counts of sgRNA were quantified by Model-based Analysis of Genome-wide CRISPR-Cas9 Knockout (MAGeCK) v5.6.0 for each sample. Count data of genes were filtered, normalized, and ranked by MAGeCK (Li et al., 2014).

### Primary Cortical Neurons Culture

Primary cortical neurons were isolated from C57BL/6 mice embryos at E17. Briefly, brains were harvested and placed in PBS/glucose where the meninges were removed and the cerebral cortices were dissected. After mechanical dissociation using sterile micropipette tips, dissociated neurons were resuspended in PBS/glucose and collected by centrifugation. Viable cells were seeded on poly-ornithine-coated 24-multiwell plate. Cells were cultured in neurobasal medium supplemented with 2 mM glutamine, 200 mM B27 supplement (Invitrogen, San Diego, CA, United States) at 37°C in a humidified incubator with 5% CO_2_. One half of the culture medium was changed every 2 days until treatment/infection. After 5 days of *in vitro* culturing, differentiated neurons were infected with lentiviral particles.

### RNA Interference

The shRNA for mouse EGLN3 gene and nontarget was obtained from Sigma Aldrich. The target sequence was 5′-CGG​CTT​CTG​CTA​CCT​GGA​CAA-3′. shRNA lentiviral particles were produced and transduced following protocols. Briefly, HEK-293T packaging cells growing in 100 mm dishes were transfected at 50–60% of confluence with a mix of 2.5 μg psPAX2 vector (packaging vector), 270 ng pMD2.G vector (envelope vector) and 2.7 μg hairpin-pLKO.1-puro-GFP vector. Lipofectamine 3000 (Invitrogen, San Diego, CA, United States) was used as transfection reagent according to the manufacturer’s instructions. After transfection, cells were cultured in high-serum medium. The media was collected after 48 h and centrifuged at 3,000 rpm at 4°C for 20 min to remove the cell debris. The supernatant was filtered (0.45-µm pore size), and concentrated by ultracentrifugation (Beckman Coulter Inc. Brea, CA, United States) at 24,000 rpm for 2 h at 4°C. Viral titres were added to the cells in the presence of 4 μg/ml polybrene (Sigma-Aldrich, St. Louis, MO, United States) and were incubated overnight for primary culture neurons. 24 h later, medium was replaced by full medium and cells were further incubated for an additional 3–4 days before testing the knockdown effects.

### Immunoblotting

Cells were washed twice with cold PBS and lysed with SDS sample buffer (63 mM Tris-HCl, 10% glycerol, and 2% SDS) containing protease and phosphatase inhibitors. Lysates were centrifuged at 14,000 g for 30 min. Supernatants were collected and measured for protein concentration. 20 µg proteins were separated on an SDS-polyacrylamide gel and immunoblotted with corresponding antibodies. The band intensity was quantified by ImageJ software (NIH, Bethesda, MD, United States).

### Immunofluorescent Staining

Cultured cells were washed twice with PBS, followed by fixation with 3.7% paraformaldehyde for 10 min and permeabilization with 0.1% Triton X-100. After blocking with 5% BSA for 30 min, cells were incubated with primary antibodies and detected with Alexa-conjugated secondary antibodies. Samples were imaged using a confocal microscope (TCS SP5; Leica, Wetzlar, Germany) with a Plan-Apochromat 63 × NA 1.4 oil differential interference contrast objective lens. The images were shown as a montage of 3 × 0.5 µm sections.

#### Detection of Cytochrome c

The cell pellets were resuspended in 10 mM Hepes (pH 7.4) containing 0.5 mM EDTA, 0.5 mM EGTA, and protease inhibitor cocktail and homogenized by passage through a 27 gauge needle. Homogenates were centrifuged for 10 min at 1,000 × g to remove insoluble material. The 1,000 × g pellet represented a crude nuclear fraction and was resuspended in the Hepes buffer containing 0.5% Triton X-100. The 1,000 × g supernatant was further centrifuged for 10 min at 18,000 × g. The 18,000 × g supernatant was used as a cytosolic fraction. Cytochrome c was determined by immunoblotting with a mouse monoclonal cytochrome c antibody (Santa Cruz Biotechnology, CA, United States).

#### Quantitative Real-Time PCR

Total RNA was isolated from the cells by using of TriZol (Invitrogen, San Diego, CA, United States). Reverse transcription was performed by using of the Reverse Transcription Kit (Promega, Madison, WI, United States). Equal amounts of total RNA (500 ng) were reverse-transcribed. Quantitative real-time PCRs were then conducted at 95°C for 10°s, followed by 40 cycles of 95°C for 5°s and 60°C for 30°s. The relative RNA levels were normalized to endogenous actin expression for each sample. The PCR experiments were repeated 3 times, each using separate sets of cultures. The primers for mouse EGLN3 is forward: 5′-CATGATTCGTCCGAGCATCC-3′and reverse: 5′-CAC​ACA​CGG​ATG​AAA​GGA​CC-3′, for Bcl-2 forward: 5′-CATGAAATCCTCCCCTCCGA-3′and reverse: 5′-AGA​ACC​CCT​GTC​TCC​AAA​GG-3.

#### Measurement of Apoptosis

Apoptosis was detected by Annexin V FITC Apoptosis Detection Kit (Sigma-Aldrich, St. Louis, MO, United States). Briefly, the cells were collected and washed twice with PBS. 500°µl binding buffer suspension was then added to the treated cells. After that, 5 µl Annexin V-FITC and 10 µl propidium iodide were added to each group and cultures were incubated at 37°C for 5∼15 min in dark. Flow cytometer (BD Biosciences, San Jose, CA, United States) was used to detect the percent cells with apoptosis and flowJo software was used for flow cytometry analysis.

### Data and Statistical Analysis

Gene enrichment terms in genes with the number of unique sgRNAs greater than three were used to further analysis. Pathway enrichment and Gene Ontology (GO) analysis was performed including “GO Molecular Function”, “GO Cellular Component”, “GO Biological Processes”, “KEGG Pathway” and “Protein-Protein Interaction (PPI)” in the web application Metascape with the default parameters (Zhou et al., 2019).

The data were presented as mean ± Standard Deviation. The significance of differences between the groups was determined by paired Student’s t-test and/or one-way ANOVA by the GraphPad Prism 6 software, with 0.05 as the level of significance.

## Results

### A Genome-Wide Clustered Regularly Interspaced Short Palindromic Repeats/CRISPR-Associated Protein 9-Mediated Screen to Identify Ammonia Resistance Genes

**FIGURE 1:**
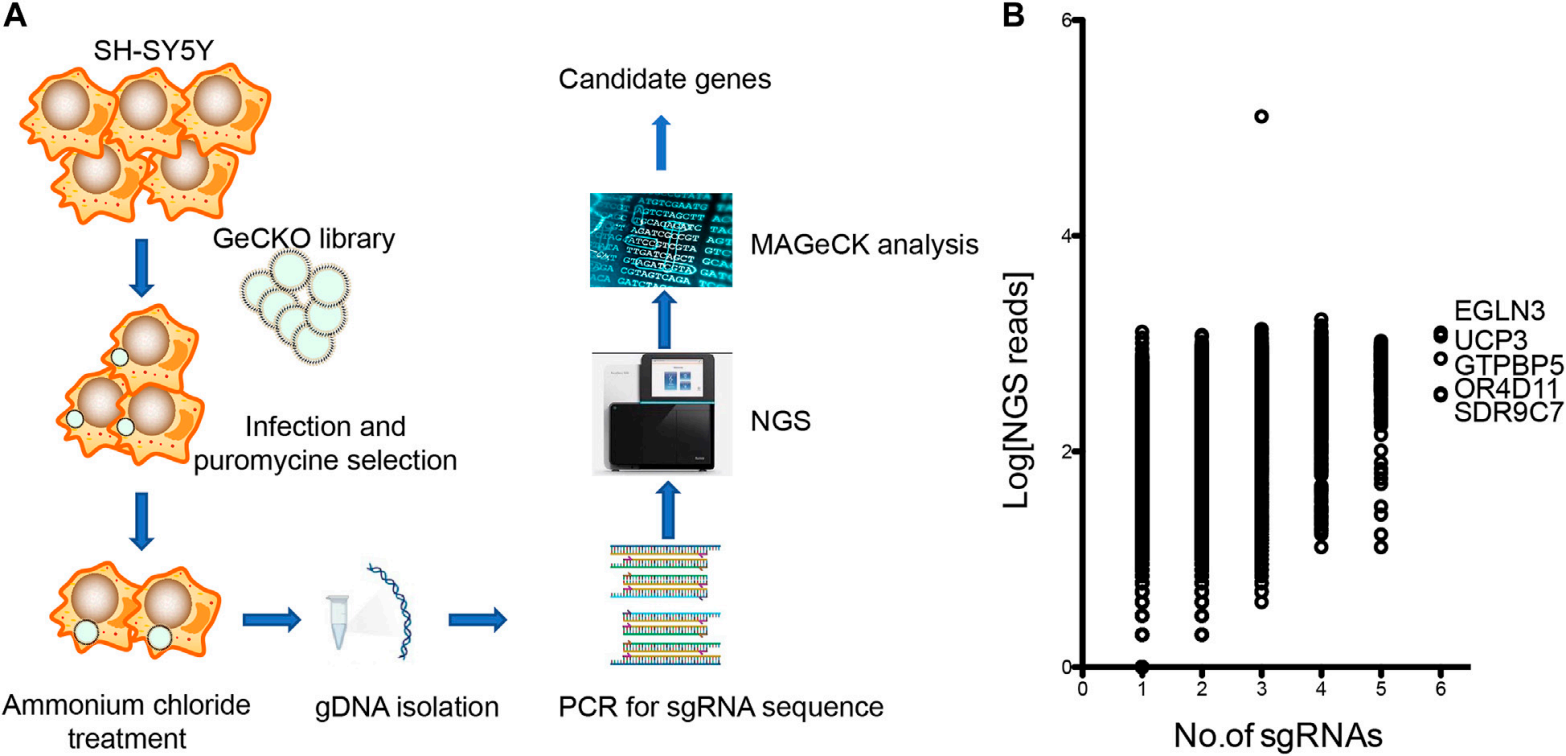
The GeCKO screen to identify genes whose loss conferred ammonia resistance. **(A)** Schematic of forward GeCKO screen in SH-SY5Y cells using pooled sgRNA libraries. **(B)** Genes identified in the screen for ammonia resistance. The X-axis was the number of unique sgRNAs for each gene. The Y-axis showed Log10 of the average of sequence reads.

CRISPR/Cas9-mediated genome-wide knockout technology provides a quick tool to study the mechanism of ammonia neurotoxicity to cells. GeCKO lentivirus library contains 123,411 sgRNAs targeting whole 19,050 human genes. A pool of cells was generated with lentivirus infection, in which every targeted gene theoretically carried a loss of function mutation in a single cell (Wang et al., 2014). Subsequently, the pooled cells were treated with ammonium chloride. Surviving cells were collected for analysis of the inserted sgRNAs in genome (Figure 1A). The sgRNAs were amplified from gDNA of collected cells. Amplicon was sequenced by next generation sequencing (NGS) and mapped to indexed library. The candidate genes were ranked depending on the number of unique sgRNAs versus NGS reads. Enrichment of candidate sgRNAs was found in our GeCKO screening, suggesting that loss of function of these genes conferred resistance to ammonia insult. Of the 19,050 genes tested, our GeCKO screen identified EGLN3, UCP3, GTPBP5, OR4D11, and SDR9C7 with 6 unique sgRNAs may contribute to protect against ammonia injury (Figure 1B, Supplementary Table S1).

### Pathway and Gene Ontology Analysis of Ammonia Resistance Genes and Construction of Protein-Protein Interaction Network

**FIGURE 2:**
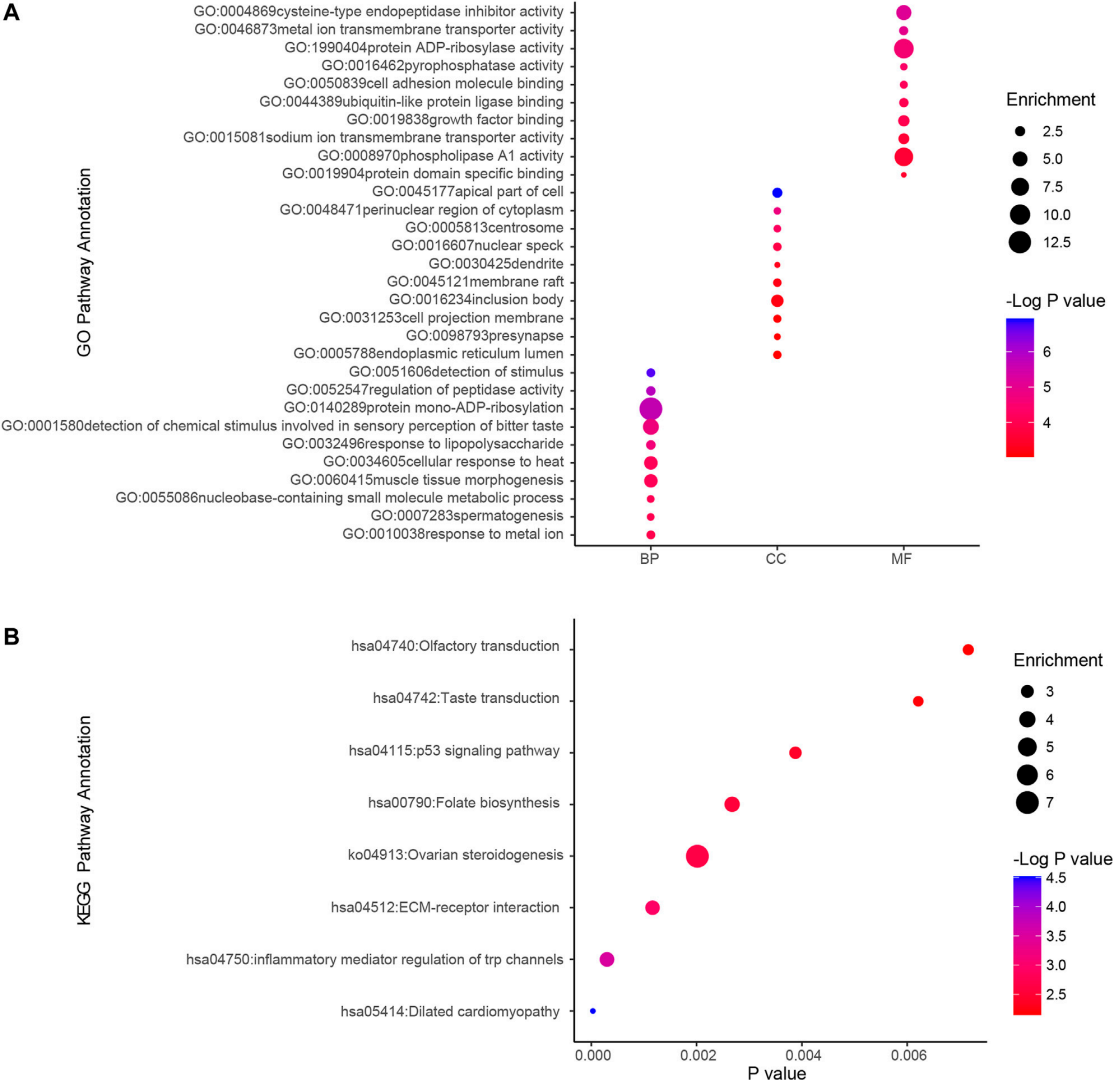
GO and KEGG cluster pathway analysis of candidate genes.Gene ontology (GO) and Kyoto Encyclopedia of Genes and Genomes (KEGG) pathway enrichment analysis by using ClusterProfiler. **(A)** GO analysis depicted the significantly enriched GO terms for top 10 enriched gene sets from candidate genes. Horizontal axis denoted different groups. The vertical axis represented strikingly enriched GO pathway names. BP, Biological Process; CC, Cellular Component; MF, Molecular Function. **(B)** KEGG analysis depicted the significantly top10 enriched GO terms for the top enriched gene sets from overlapping genes. The horizontal axis represented the respective −Log10 (corrected *p*-value) of distinct pathways relative to the other displayed terms. The vertical axis represented considerably enriched KEGG pathway names. The color represented the *p*-values relative to the other displayed terms (Blue was more meaningful). Size of round node was in proportion to gene ratio of the enriched gene number.

We used GO analysis and KEGG analysis of candidate genes to understand the possible molecular mechanisms of ammonia neurotoxicity. The top 10 significant pathways and functions were listed in accordance with *p* values and proportion to gene ratio of the enriched gene number (Figure 2). The GO analysis of Biological Process (BP), Cellular Component (CC) and Molecular Function (MF) demonstrated that ammonia resistance genes were primarily involved in cysteine-type endopeptidase inhibitor activity, metal ion transmembrane transporter activity, protein ADP-ribosylase activity, apical part of cell, perinuclear region of cytoplasm, centrosome, detection of stimulus and regulation of peptidase activity (Figure 2A). KEGG cluster pathway analysis of candidate genes showed inflammatory mediator regulation of trp channels and ECM-receptor interaction may be involved in ammonia neurotoxicity (Figure 2B).

**FIGURE 3:**
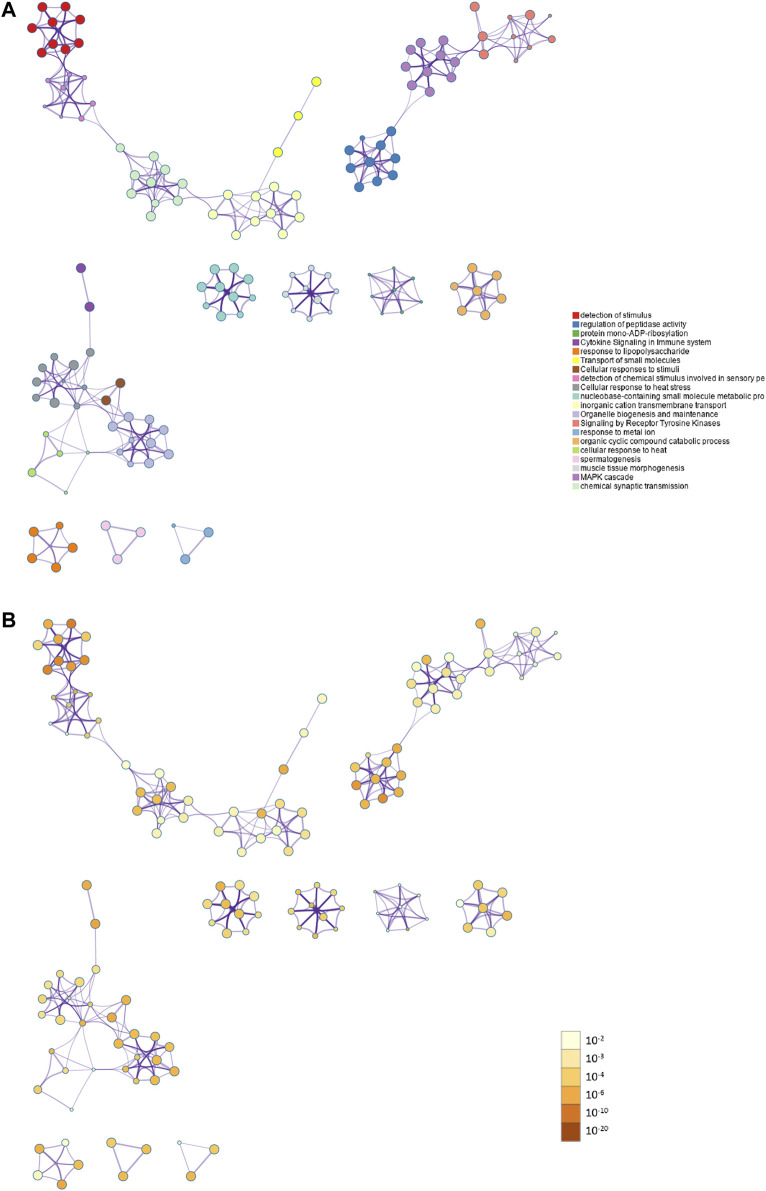
Action view from the PPI network analysis of the candidate genes. **(A)** The top 20 significantly enriched biological process and pathways related to ammonia resistance genes with network. Different colors in the map represented different function groups. **(B)** The same enrichment network had its nodes colored by *p*-value. The darker the color, the more statistically significant the node was.

PPI enrichment analysis was performed by applying Metascape to better understanding the interaction among ammonia resistance candidate genes. MCODE algorithm was used to identify densely connected network components. The biological functions of the MCODE components included detection of chemical stimulus involved in sensory pe, signaling by receptor tyrosine kinases, MAPK cascade, chemical synaptic transmission. Schematic representation of PPI network components in the PPI network was shown in Figure 3.

### Effect of Egl-9 Family Hypoxia-Inducible Factor 3 From the Genome-Scale CRISPR-Cas9 Knockout Screen on Ammonia-Induced Apoptosis in Neurons

**FIGURE 4:**
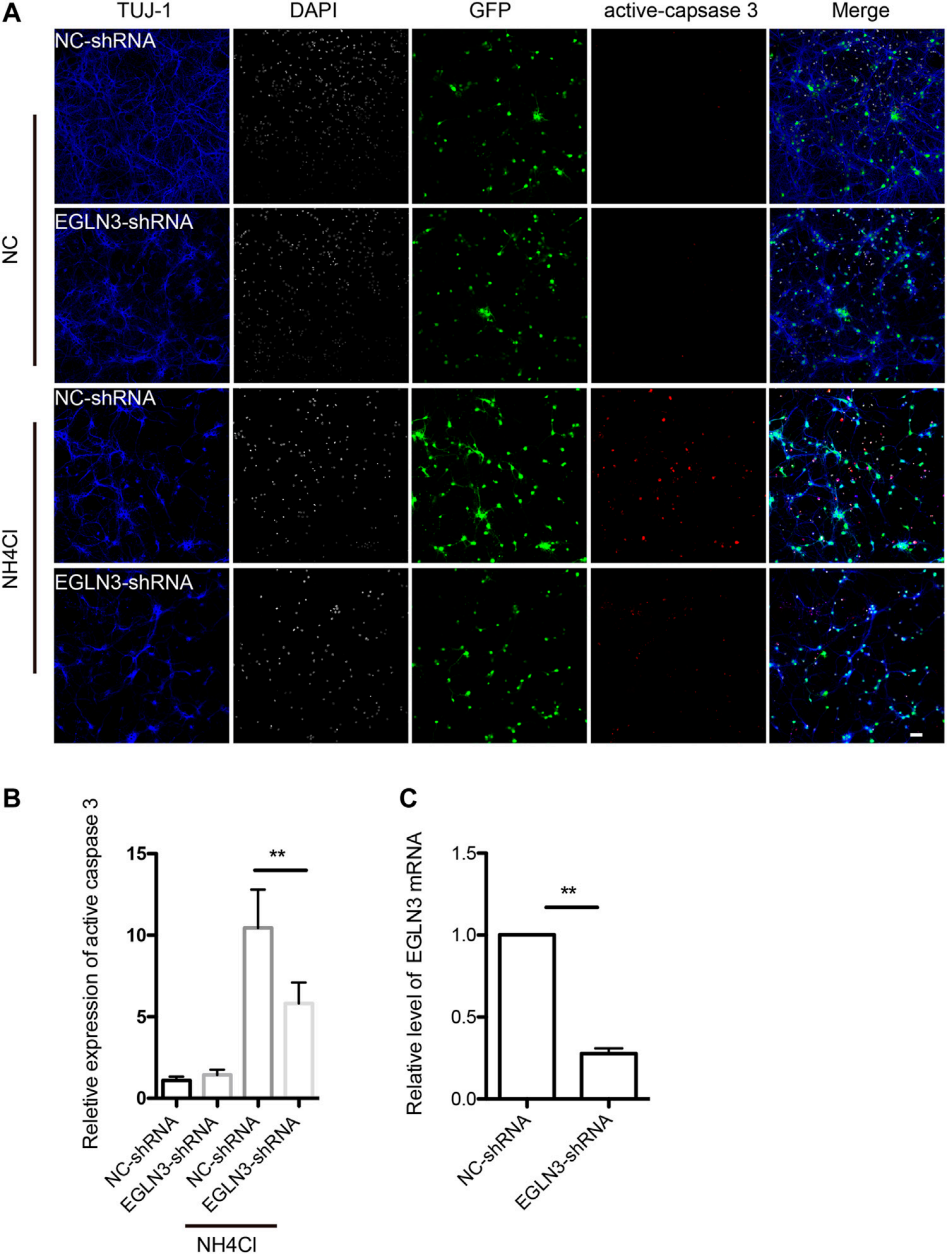
EGLN3 knockdown decreased ammonia-induced neuronal apoptosis.Neurons were infected with shRNA lentivirus (GFP green) targeting EGLN3 or control shRNA lentivirus and subjected to ammonia treatment. **(A)** Immunofluorescent stain using antibodies against neuron-specific β-tubulin TUJ 1 (blue color), activated caspase-3 (red color), and counterstain with 4,6-diamidino-2-phenylindole (white color) to show nuclei revealed EGLN3 inhibition decreased activated caspase-3 expression during ammonia treatment. **(B)** Quantitation (mean ± SEM) of **(A)** from three independent experiments. Ammonia induced activated caspase-3 expression was attenuated by EGLN3 knocked-down. **(C)** EGLN3 knockdown efficiency was validated by quantitative real-time PCR. **p < 0.01. bar = 10 µm.

We selected EGLN3 with 6 unique sgRNAs in GeCKO screening for verification. Our results confirmed that EGLN3 affected cell apoptosis induced by NH_4_Cl treatment. We knocked down EGLN3 by infecting neuron with specific shRNA lentivirus (Figure 4C). Expression of activated caspase-3 was analyzed in neurons infected with shRNA lentivirus or control after 5 mM NH_4_Cl exposure. We observed a significant decrease in activated caspase-3 expression in neurons with NH_4_Cl treatment following knockdown of EGLN3 (Figures 4A,B).

### Egl-9 Family Hypoxia-Inducible Factor 3 Knockdown Ameliorated Ammonia Induced Mitochondrial Apoptosis Activation in Neurons

**FIGURE 5:**
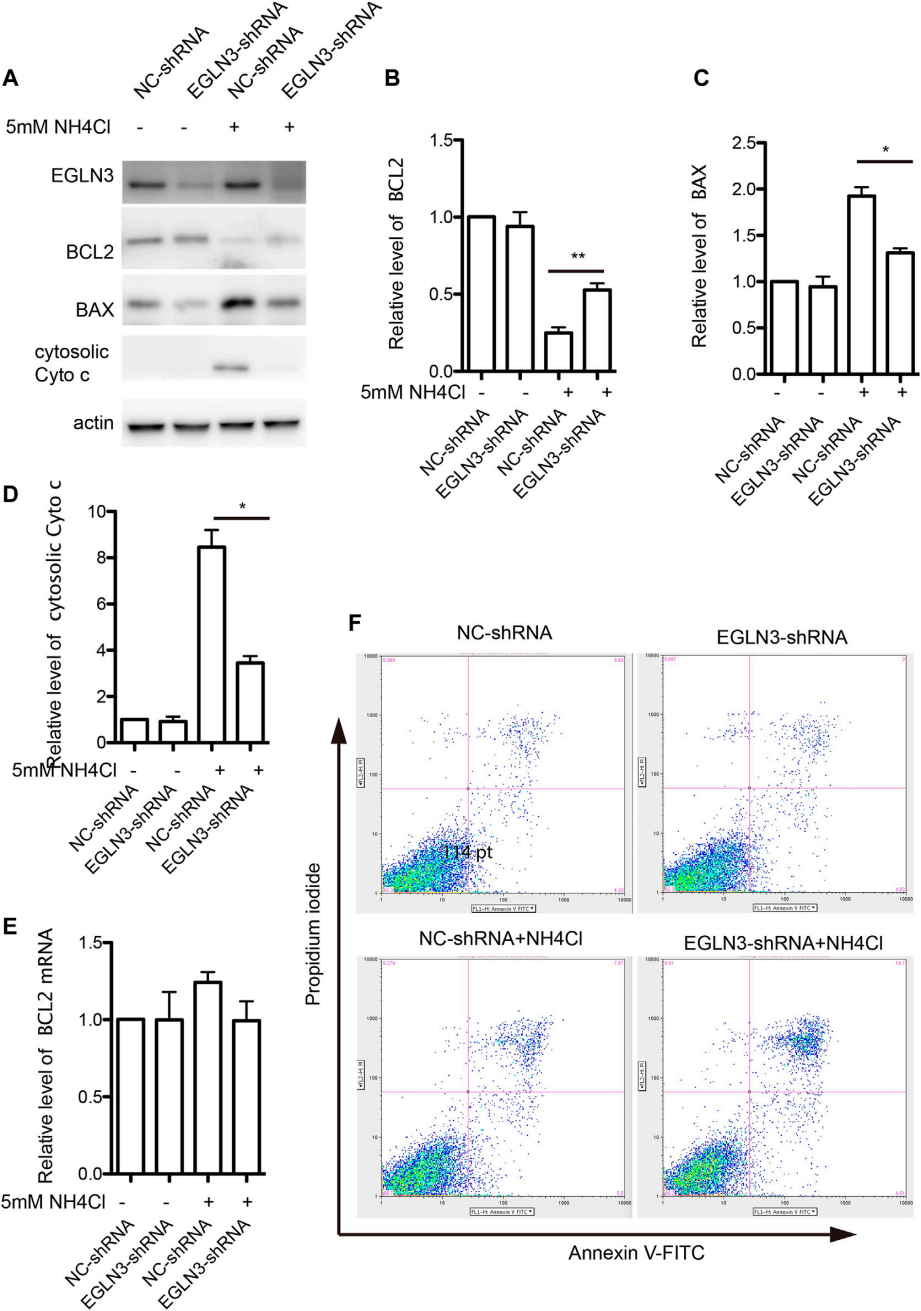
EGLN3 inhibition attenuated mitochondrial apoptosis activation induced by NH_4_Cl exposure. **(A)** Western blot was performed to examine the protein levels of mitochondrial apoptosis proteins Bcl-2, Bax and cytosolic cytochrome c in neurons subjected to NH_4_Cl insult. Actin was as a loading control. EGLN3 knockdown significantly attenuated ammonia induced down-regulated expression of Bcl-2, up-regulated expression of Bax and release of cytochrome c into the cytosol. **(B)** Quantitation (Mean ± SEM) of Bcl-2 expression from three independent experiments. EGLN3 inhibition ameliorated decreased expression of Bcl-2 during NH_4_Cl exposure. **(C)** Quantitation (Mean ± SEM) of Bax expression from three independent experiments. EGLN3 inhibition ameliorated increased expression of Bax during NH_4_Cl exposure. **(D)** Quantitation (Mean ± SEM) of cytochrome c expression from three independent experiments. EGLN3 inhibition ameliorated release of cytochrome c into the cytosol. **(E)** Quantitative real-time PCR was performed to examine the mRNA level of Bcl-2. EGLN3 knockdown has no effect on the expression of Bcl-2 mRNA with or without NH_4_Cl insult. **(F)** EGLN3 inhibition significantly decreased NH_4_Cl treatment induced apoptosis by flow cytometry with Annexin V-FITC/propidium iodide staining. *p < 0.05, **p < 0.01.

Bcl-2, Bax and cytochrome c are important mitochondrial apoptosis proteins, while Bcl-2 promotes cell survival and Bax accelerates apoptosis. Western blotting results revealed that the expression of Bcl-2 was significantly decreased in neurons treated with NH_4_Cl, which was attenuated by knockdown of EGLN3. However, EGLN3 knockdown had no effect on the expression of Bcl-2 mRNA (Figures 5A,B,E). However, the expression of Bax was significantly increased after NH_4_Cl exposure, which was rescued by infection of EGLN3-shRNA lentivirus (Figures 5A,C). The release of cytochrome c from the mitochondria into the cytosol is a trigger for apoptosis. EGLN3 knockdown also significantly decreased ammonia induced release of cytochrome c into the cytosol (Figures 5A,D).

We further verified EGLN3 knockdown on ammonia-induced apoptosis by flow cytometry with Annexin V-FITC/propidium iodide staining. As shown in Figure 5F, knockdown of EGLN3 also significantly decreased NH_4_Cl treatment induced apoptosis.

## Discussion

HE significantly impairs patients’ quality of life and brings great burdens to their families and the whole society. However, the precise pathogenesis of HE is still not fully clarified and effective therapeutic options are urgently needed (Patidar and Bajaj, 2015; Wijdicks, 2016). Among the multiple factors that are supposed to contribute to the pathogenesis of HE, ammonia neurotoxicity is one of the most prevalent and recognized hypothesis (Hadjihambi et al., 2018; Butterworth, 2019). Therefore, we performed a whole genome-scale CRISPR-Cas9 loss of function selection screen in SH-SY5Y cells to discover potential contributors to ammonia neurotoxicity. We also used GO analysis, KEGG analysis and PPI enrichment analysis of candidate genes to understand the possible molecular mechanisms of ammonia neurotoxicity. Our results suggested that EGLN3, UCP3, GTPBP5, OR4D11, and SDR9C7 may contribute to protect against ammonia neurotoxicity, while EGLN3 may be most related to ammonia resistance.

EGLN3 (egl-9 family hypoxia-inducible factor 3), also termed PHD3, HIFPH3 or HIFP4H3, is a member of the EGLN family of prolyl hydroxylases. EGLN3 catalyzes the hydroxylation of the α subunits of hypoxia-inducible factor and inhibits the NF-κB signaling pathway (Fu, 2016). In our study, we confirmed that EGLN3 knockdown by shRNA decreased ammonia induced caspase-3 activation. EGLN3 promotes apoptosis under normoxia and the activity of EGLN3 is necessary for apoptosis induction (Jaakkola and Rantanen, 2013). EglN3 promotes apoptosis through regulation of the kinesin KIF1Bbeta (Schlisio et al., 2008). During hypoxia, the activity of EGLN3 is inhibited, which contributes to hypoxia induced apoptosis resistance and cell survival promotion (Henze et al., 2014; Li et al., 2019). EGLN3 inhibition also decreased apoptosis induction during ammonia insult, suggesting that EGLN3 is a potential therapeutic target for ammonia induced neurotoxicity in HE.

Many neuroprotective agents attenuate neuronal injury by targeting EGLN3. miR-451 confers neuroprotection in cerebral ischemic injury through regulation of EGLN3 (Wang et al., 2021). However, more efforts are still needed to better clarify the underlying molecular mechanisms of EGLN3 in ammonia induced apoptosis to pave way for developing novel effective treatment. c-Jun acts upstream of EGLN3 and SDH activity is required for the proapoptotic activity of EGLN3 (Lee et al., 2005). EGLN3 knockdown leaded to ATR/CHK1/p53 signaling pathway inactivation and suppressed DNA damage induced apoptosis (Xie et al., 2012). In our study, we found that EGLN3 knockdown increased Bcl-2 expression, decreased expression of Bax and release of cytochrome c into the cytosol after ammonia injury, suggesting that EGLN3 inhibition protects against ammonia induced apoptosis through mitochondrial dependent apoptosis pathway. Mitochondrial dysfunction is one of the pivotal causes of ammonia induced neurotoxicity. Neuronal mitochondria protection is supposed to be a promising strategy for the treatment of HE (Heidari, 2019). Sirtuin-3 activation confers neuroprotection in a rat model of HE through attenuating ammonia induced mitochondrial damage and maintaining mitochondrial integrity of hippocampal neurons (Anamika and Trigun, 2021). Our data found that EGLN3 inhibition protected against ammonia induced apoptosis by targeting mitochondria, which will lay the foundation for future therapeutic strategies by regulating EGLN3.

In summary, we have successfully applied a genome-scale CRISPR-Cas9 screen in SH-SY5Y cells to identify ammonia resistance genes. We have identified EGLN3, UCP3, GTPBP5, OR4D11 and SDR9C7 may contribute to protect against ammonia injury. We confirmed that EGLN3 knockdown decreased ammonia-induced neuronal apoptosis, which is through mitochondrial dependent apoptosis pathway. Our study will pave way for better understanding of the molecular mechanisms underlying ammonia neurotoxicity and developing new therapeutic options for HE.

## Data Availability

The original contributions presented in the study are publicly available. This data can be found here: https://www.ncbi.nlm.nih.gov/sra/?term=SRR18148900.
